# Identification, Elucidation and Deployment of a Cytoplasmic Male Sterility System for Hybrid Potato

**DOI:** 10.3390/biology13060447

**Published:** 2024-06-18

**Authors:** Ernst-Jan Eggers, Ying Su, Esmee van der Poel, Martijn Flipsen, Michiel E. de Vries, Christian W. B. Bachem, Richard G. F. Visser, Pim Lindhout

**Affiliations:** 1 Solynta, Dreijenlaan 2, 6703 HA Wageningen, The Netherlandschristian.bachem@wur.nl (C.W.B.B.); 2 Plant Breeding, Wageningen University & Research, P.O. Box 386, 6700 AJ Wageningen, The Netherlandsrichard.visser@wur.nl (R.G.F.V.); 3 Graduate School Experimental Plant Sciences, Wageningen University & Research, 6708 PB Wageningen, The Netherlands; 4 Hogeschool Arnhem Nijmegen, Laan van Scheut 2, 6525 EM Nijmegen, The Netherlands

**Keywords:** diploid potato breeding, cytoplasmic male sterility, QTL analysis, berry and seed production

## Abstract

**Simple Summary:**

Conventional potato breeding has produced only limited genetic gain due to the polyploid nature of the crop. In recent years, hybrid potato breeding at the diploid level has been developed to overcome this limited genetic gain. In diploid potato breeding, homozygous inbred lines are developed by self-fertilization, enabling incremental improvements of the material in each generation. This type of breeding requires self-fertility, which makes hybridization of inbred lines labor-cumbersome and results in hybrids that produce many undesirable berries in the field. In many crop species, cytoplasmic male sterility is used to produce maternal inbred lines that are male sterile. In this study, we explore the antherless cytoplasmic male sterility system in potato. We identify a recessive locus that is required for sterility and we show that this trait is expressed in *Phureja* cytoplasm but not in *Andigena* or *Tuberosum* cytoplasm. We implemented this system in hybrid seed production and show that the resulting hybrids set far fewer berries in the field than male fertile controls.

**Abstract:**

Recent advances in diploid F_1_ hybrid potato breeding rely on the production of inbred lines using the *S-locus inhibitor* (*Sli*) gene. As a result of this method, female parent lines are self-fertile and require emasculation before hybrid seed production. The resulting F_1_ hybrids are self-fertile as well and produce many undesirable berries in the field. Utilization of cytoplasmic male sterility would eliminate the need for emasculation, resulting in more efficient hybrid seed production and male sterile F_1_ hybrids. We observed plants that completely lacked anthers in an F_2_ population derived from an interspecific cross between diploid *S. tuberosum* and *S. microdontum*. We studied the *antherless* trait to determine its suitability for use in hybrid potato breeding. We mapped the causal locus to the short arm of Chromosome 6, developed KASP markers for the *antherless* (*al*) locus and introduced it into lines with T and A cytoplasm. We found that *antherless* type male sterility is not expressed in T and A cytoplasm, proving that it is a form of CMS. We hybridized male sterile *al/al* plants with P cytoplasm with pollen from *al/al* plants with T and A cytoplasm and we show that the resulting hybrids set significantly fewer berries in the field. Here, we show that the antherless CMS system can be readily deployed in diploid F_1_ hybrid potato breeding to improve hybridization efficiency and reduce berry set in the field.

## 1. Introduction

In recent years, potato (*Solanum tuberosum*) breeding has seen a shift towards breeding on the diploid level [1,2,3,4,5,6]. Conventional potato breeding is usually performed at the tetraploid level, where two heterozygous clones are crossed and selections take place among large numbers of F_1_ offspring. It requires many locations and years (10–15 years) of testing to select a single commercial cultivar and genetic gain is relatively low [6,7,8]. The push towards inbred line-based diploid F_1_ hybrid breeding is expected to speed up the breeding process and improve genetic gain by allowing continuous improvement of inbred lines. In addition, bulking up a new cultivar is fast as millions of hybrid seeds can be produced per year. Elite inbred lines can be further improved by introgression of favorable genes, such as resistance genes, by means of a backcrossing program [9].

To allow inbreeding of self-incompatible diploid potato clones, these clones can be crossed with a line containing the dominant allele of the *Sli* (*S-locus inhibitor*) gene, which enables self-fertilization [10,11,12]. The *Sli* gene, which encodes an F-box PP2-B10 protein, is expressed in pollen and interacts with the maternal components of the gametophytic self-incompatibility (GSI) system, the S-RNases, during pollination, leading to a breakdown of the GSI system [13,14]. From a breeding point of view, this system works well and has resulted in the production of inbred lines in multiple programs [4,5,6,15,16,17,18]. However, as a consequence of the presence of *Sli*, maternal inbred lines are self-fertile, which necessitates emasculation for hybrid seed production. The manual emasculation process takes time and may lead to reduced hybrid seed set due to the mechanical stress imposed on the flower buds. Another, more detrimental, effect is that the resulting F_1_ hybrid offspring are self-fertile as well. While F_1_ hybrid fertility is essential in crops where seeds are the end product, for potato, where the tubers are the end product, this is not the case. Primarily, this is because the seeds in the berries can remain viable in the field and produce a volunteer crop, which may act as a reservoir for diseases and disrupt crop rotation schemes [19]. Additionally, the growth of these berries and seeds may sequester photo-assimilates that might otherwise have been directed to tuber bulking [20]. A straightforward route to resolve these problems would be to introduce cytoplasmic male sterility (CMS) to the maternal lines as is commonly performed in many F_1_ hybrid crops [21]. CMS systems exploit incongruities between organellar genomes and nuclear genomes, which lead to a failure to produce fertile pollen.

Sterilizing cytoplasms have been identified in many crop species, like rice, wheat, soybean, sunflower, rapeseed and several more [21]. CMS has been successfully implemented in breeding programs [22,23,24,25,26]. However, deployment of CMS in crops has not always gone smoothly. For instance, CMS has been associated with deleterious effects such as susceptibility to southern corn blight in maize and the inefficiencies of pollen donors or insect vectors in soybean [27,28]. In crop species where the end product is seed, implementations of CMS rely on the *three-line system* in which a *CMS* line is maintained by an isogenic *maintainer* line and male fertility in the hybrid is restored by crossing the CMS line to the *restorer* [29,30,31,32]. In potato, the end product is the belowground tubers, and restorer lines are not required. In fact, a major goal of implementing CMS in hybrid potato breeding would be to avoid fertility in the F_1_ hybrid to ensure minimal berry and seed production in the field.

Among potato and its wild relatives, many cytoplasm types are present. In a study of cultivated potato and closely related wild species, Hosaka and Sanetomo distinguished 129 chloroplast DNA (ctDNA) types and 63 mitochondrial DNA (mtDNA) types using RFLP, CAPS, SSR and SCAR markers, resulting in 164 unique cytoplasm types [33]. Hosaka and Sanetomo proposed to group these 164 cytoplasm types into 6 types (T, D, P, A, M and W). Cytoplasm types T, D and W have been associated with male sterility in multiple breeding populations, whereas P and A cytoplasm are usually associated with male fertility [34,35,36,37,38,39]. Recently, Santayana et al. observed segregation for male fertility among CIP potato breeding populations with T and D cytoplasm and identified parental lines that potentially contain nuclear *Restorer of Fertility* (*Rf*) genes for D and T cytoplasm [40]. On the cytoplasmic side, Sanetomo et al. identified a recombinant mitochondrial DNA molecule, RC-I, the presence of which is completely associated with tetrad sterility (T-CMS) among interspecific hybrids derived from *S. stoloniferum* Schltdl. & Bouché [41]. Interestingly, T-CMS must be the result of an interaction between a dominant *S. tuberosum* nuclear gene and the mitochondrial RC-I molecule, since hexaploid interspecific hybrids containing the complete *S. stoloniferum* genome still show T-CMS and thus should contain all dominant *Rf* genes present in the *S. stoloniferum* genome [42]. For the present study, only three cytoplasm types are relevant: A, P and T cytoplasm, originating from *S. tuberosum* ssp *andigenum* Hawkes, *S. phureja* Juz. et Buk. and *S. tuberosum*, respectively.

While most genotypes with P cytoplasm are male fertile, Endelman and Jansky observed segregation for anther length in an F_2_ derived from the cross DM × M6, whereby DM carries the P cytoplasm. In this population, 23% of the progeny had short anthers (SA) that did not shed any pollen. QTL analysis revealed a single recessive allele (*sa*) on the short arm of Chromosome 6 to be responsible for this SA phenotype. Interestingly, the recessive allele was inherited from the male fertile M6 genotype whose self-fertilized progeny do not show the SA phenotype, leading the authors to suggest that the SA phenotype is the result of an interaction between the recessive *sa* allele from M6 and the P cytoplasm from DM [43]. At Solynta, we observed complete absence of anthers (which we named antherless) in F_2_ genotypes derived from an interspecific hybrid *S. tuberosum* × *S. microdontum* subs. *gigantophyllum*. The antherless phenotype is similar to the short anther phenotype, except that the level of malformation varies, ranging from complete absence of anthers to somewhat malformed anthers. Given the importance of CMS for diploid hybrid potato breeding, we set out to elucidate the genetics and applicability of the antherless trait. We aimed to localize the causal locus in the nuclear genome via QTL analysis and to determine whether it is a form of CMS by introducing the trait to lines with non-P cytoplasm types. Here, we report on the mapping of the causal gene and the characterization of this CMS system for fertility traits. Proof of principle was obtained by exploiting this CMS system to produce male sterile maternal lines and hybrids.

## 2. Materials and Methods

### 2.1. Plant Materials

An overview of all plant materials used in this study is available in Table S1. All *S. tuberosum* genotypes used in this study are derived from the founders of the Solynta breeding program as described in Lindhout et al. (2016) [1]. The donor of the antherless trait was derived from a CGN accession of *S. microdontum* subs. *gigantophyllum*, which is available from CGN (Wageningen, The Netherlands) under accession number CGN18200. *S. microdontum* subs. *gigantophyllum* is a potato wild relative with resistance against late blight and wart disease [44,45]. It is diploid and has an endosperm balance number of 2 and is therefore crossable with diploid *S. tuberosum*.

### 2.2. Crossing Conditions

To avoid any unwanted out-crossing and control plant growth as best as possible, experiments involving crossing were conducted in a greenhouse. Greenhouse conditions were the same as described in Eggers et al. (2021) [13].

### 2.3. Phenotypic Analysis of Male Fertility

**Figure 1:**
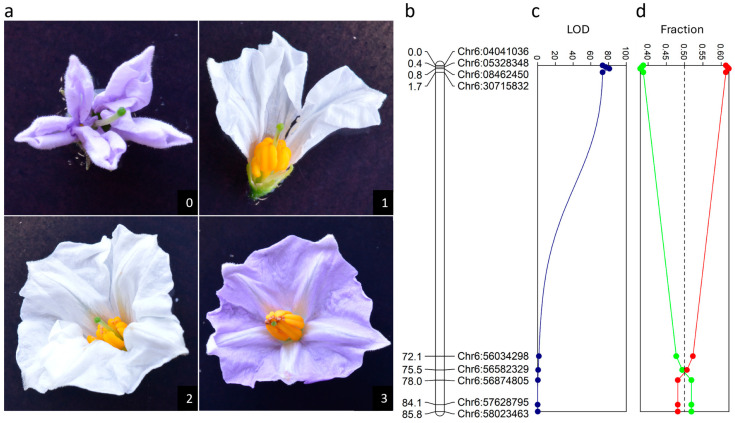
Population BC2(P)-1 segregates for anther length. (**a**) Anther phenotypes in BC_1_ population BC2(P)-1 scored from 0 (complete absence of anthers) to 3 (normal anthers). (**b**) Genetic map of Chromosome 6. (**c**) QTL analysis for anther score reveals a significant QTL on the top of Chromosome 6. (**d**) Segregation distortion on the top of Chromosome 6 reduces the number of antherless plants. In red, the fraction of plants heterozygous per locus is shown and in green the fraction of plants homozygous for the antherless donor allele is shown.

Anther malformation was assessed on a scale from 0 to 3, where a score of 0 means complete absence of anthers and a score of 3 means normal anthers (Figure 1a). Flowers with anthers (scores 1–3) were vibrated using an electronic toothbrush while collecting the pollen in a micro-centrifuge tube. The amount of pollen shed was scored on a scale from 0–3 (where a score of 0 means no pollen shed, and a score of 3 means abundant pollen shed). Self-pollinations were made using the collected pollen, and the micro-centrifuge tubes with leftover pollen were placed in sealed containers with abundant silica gel beads to dry. To assess the viability of the pollen, one drop of acetocarmine was added to the pollen and incubated for one minute. The samples were then vortexed and 10 µL of the mix were pipetted into a counting chamber and observed with normal light microscopy. Stained and unstained pollen were counted and viability was scored using the formula: $Viable\;pollen\;(\%)=\frac{Stainable\;pollen}{Total\;pollen}\times 100$.

### 2.4. SeqSNP Genotyping, Linkage Analysis and QTL Mapping of Population BC2(P)-1

Leaf discs from 249 individuals of population BC2(P)-1 were sampled in 96-well plates and were genotyped via SeqSNP™ (LGC Genomics GmbH, Berlin, Germany) [46]. SNPs were extracted from the SeqSNP™ reads using the method described in Adams et al. (2023) [47], resulting in 2116 SNPs. SNPs that were homozygous alternate between F2-1 and Solyntus were selected. SNPs with more than 10% missing data and duplicate SNPs originating from the same SeqSNP™ probes were removed, resulting in a genotypic dataset of 244 SNPs (Table S2). This dataset was converted to Joinmap coding and genetic maps for all 12 chromosomes were created using Joinmap 4.1 [48] with population type BC1 and default settings (Table S3). QTL mapping was performed using interval mapping in MapQTL6 [49].

### 2.5. KASP Marker Development and Genotyping

Between 30 and 50 mg of leaf material was collected from genotype F2-1 and submitted for DNA extraction, library preparation and Illumina PE150 sequencing with 30× coverage by Novogene UK Company LTD (Cambridge, UK). The reads were mapped to DM4.03 with BWA and variants were called using BCFtools. Several thousand high-quality variants in F2-1 and Solyntus were inspected in jBrowse to determine suitability for KASP genotyping. For KASP genotyping, leaf discs from population BC2(P)-1 were sampled in 96-well plates and submitted to VHLgenetics (Wageningen, The Netherlands) for DNA extraction and KASP analysis as described in Eggers et al. (2021) [13]. The quality of the resulting KASP marker data was assessed using SNPviewer (lgcgroup.com/products/genotyping-software/snpviewer); markers that did not segregate or showed unexpected segregation were discarded from further analysis. For the remaining markers, segregation ratios were tested using χ2 tests with the null hypothesis that the antherless locus segregates in mendelian 1:2:1 ratio. *p*-values were calculated from the χ2 to determine the likelihood that the null hypothesis of mendelian 1:2:1 segregation is true.

### 2.6. Field Experiment

The field trial was designed with plots of four ridges of 32 plants per plot with 8 plants per ridge and with plots replicated in two randomized blocks. The plants were spaced at 25 cm on each ridge, and the distance between ridges was 75 cm. Plots were separated by an empty row, one row of cultivar Bergerac, and then another empty row. The design included the 13 antherless proof-of-concept hybrids and three male fertile hybrids as controls. The seeds were sown and raised in a greenhouse and transplanted six weeks after sowing to a field in Heelsum, The Netherlands on the 17th of May 2023. Crop handling was performed according to standard agricultural practices, as described by Kacheyo et al. (2023) [50]. At 108 days after transplanting, we harvested all berries from the plants on the middle two ridges of each plot, including the berries that were already detached from the plants but were present between the two middle ridges. All berries were bulked per plot in a mesh bag and fresh weight was determined and used for analysis. The significance of the difference in berry weight per plant between the *antherless* plants and male fertile controls was determined using a *t*-test. Two datapoints with more than two times the standard deviation from the mean among the antherless plants were removed for the *t*-test.

## 3. Results

### 3.1. Identification of the Antherless Phenotype and Development of a Mapping Population

In a breeding program at Solynta, we identified plants lacking anthers in an F2 population derived from a cross between *S. tuberosum* × *S. microdontum* subs. *gigantophyllum* (F2-1). We designated this phenotype “*antherless*”. In this small population of 50 plants, we identified three individuals that completely lacked anthers (Figure 1a, score = 0), suggesting that the phenotype is caused by two recessive loci or one locus with severely skewed inheritance. We set out to characterize the loci that are responsible for the antherless phenotype, to elucidate the genetics and to determine the contribution of cytoplasm types. To generate a dedicated mapping population, we pollinated one antherless plant, F2-1, with pollen from the *S. tuberosum* genotype Solyntus [51]. In the resulting BC_1_ population BC1(P), all plants had normal anthers, confirming the recessive nature of the antherless gene(s). To study this locus in more detail, we backcrossed BC1(P)-1 to F2-1 to generate population BC2(P)-1.

### 3.2. Segregation of the Antherless Phenotype in Population BC2(P)-1

In the BC2(P)-1 population, we distinguished four anther phenotypes, complete absence of anthers (score 0), severely reduced anthers (score 1), moderately reduced anthers (score 2) and normal anthers (score 3) (Figure 1a). Plants that lacked anthers or showed reduced anthers (scores 0–2) never released any pollen upon vibration with an electric toothbrush. We grew 252 individuals from this BC2 population in the greenhouse and obtained 225 flowering plants (Table S4). We identified 84 genotypes which lacked or had reduced anthers, whereas the remaining 141 plants all had normal anthers. Among the 84 plants with malformed anthers, 32 showed complete absence of anthers, 35 had severely reduced anthers, and 17 plants had moderately reduced anthers. We genotyped the population using SeqSNP, resulting in 656 segregating markers. After removing non-informative markers, we generated a map with 153 SNPs covering 12 chromosomes which we used for QTL mapping of the anther phenotype. We found one highly significant QTL on the top of Chromosome 6 (LOD = 80.92 Figure 1c). Interestingly, other flower-related traits, such as number of floral buds, bud abortion and corolla shape, mapped to this locus as well (Table S5). Further inspection of individual recombinants showed that the causal locus is located in a 25.12 Mb interval between 5.59 and 30.71 Mb on the reference genome DM6.1 [52]. All plants that are heterozygous in this interval have normal anthers (score = 3), whereas those individuals that are homozygous for the *S. microdontum* haplotype all have reduced or absent anthers (score = 0, 1 or 2), indicating that in this population, antherless is effectively a monogenic recessive trait (Figure 1c). This locus is designated *Al*. We observed significant segregation distortion at the top of Chromosome 6, where the *Al* locus is located (χ2 = 14.33, *p* < 0.001) (Figure 1d).

To reduce the size of the interval, we screened 2011 new seedlings from a closely related BC2 (BC2(P)-2). We screened this population for recombination in the interval using four KASP markers, two of which were located at the proximal side and the other two located at the distal side of the 25.12 Mb interval. We identified only 16 putative recombinants in this region. As the number of these putative recombinants compared to the size of the population (n = 2011) was low, these results could also be explained by some rare errors in the marker analyses. Therefore, we genotyped these 16 putative recombinants with 25 additional KASP markers. Indeed, the genotypic data of these additional markers showed that 2 of the 16 putative recombinants had a recombination just outside the interval at the centromeric side, whereas the other 14 likely resulted from genotyping errors in the initial genotyping with the flanking markers (Table S6).

### 3.3. Expression of the Antherless Phenotype in A and T Cytoplasm Types

The results so far show that homozygosity for the recessive *al* allele leads to the antherless phenotype in material with P cytoplasm. However, to apply the antherless system in breeding for male sterile hybrids, it is important to know whether it is a form of CMS, and we set out to introduce the *al* allele in lines with different cytoplasm types. We selected one clone from the proprietary Solynta genebank, D02, that has the A-type cytoplasm, and D03, D12 and two dihaploids from cultivar VR808 that have the T-type cytoplasm. These cytoplasm types were identified using pedigree analyses and were confirmed by using the multiplex PCR markers as described by Hosaka and Sanetomo [53] (Figure S1). We crossed these five cytoplasm type genitors as females with BC1-1 (*Alal*) and used six *al*-specific KASP markers to identify BC_2_ plants that received the recessive *al* allele from BC1-1. We selected eight self-fertile *Alal* heterozygous BC_2_ plants and self-fertilized these to proceed to the BC_2_F_2_ generation. The BC_2_F_2_ populations were genotyped with four KASP markers in the interval to determine the genotype at the *Al* locus. Interestingly, we observed distorted segregation in both directions; two populations, BC2F2(A)-1 and BC2F2(A)-2, showed significant distortion towards the *Al* allele, five populations showed no significant distortion, and one population showed significant distortion in favor of the *al* allele (Table 1). From all eight populations, we selected all plants that were homozygous for the *antherless* haplotype (*alal*) and transplanted these to pots. For the populations for which we had fewer than 25 *alal* plants, we added *Alal* and *AlAl* plants to fill up to at least 25 genotypes per population, allowing us to compare the effects of the genotype of the *Al* locus on the anther phenotype (Table S7).

**Figure 2:**
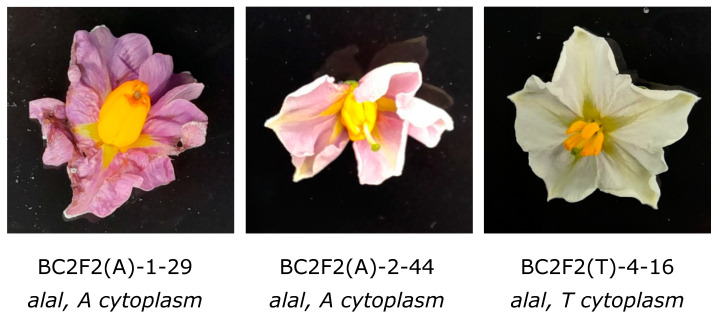
Flowers of alal homozygous genotypes with A and T cytoplasm have anthers that produce pollen and set self-seed.

Generally, we observed poor fertility in the F_2_ populations, with 80 of the 200 BC_2_F_2_ plants not producing any open flowers. Among the flowering F_2_ plants, anther morphology segregated, ranging from severely reduced anthers to normal anthers, which we scored using the same 0–3 scale. Pollen release segregated as well, with many plants not releasing any pollen at all. Interestingly, the level of anther malformation and pollen release was independent of the genotype of the *Al* locus, suggesting that factors other than the antherless locus cause reduced fertility in these populations. Most importantly, from the 120 flowering F_2_ plants, 8 produced seed after self-pollination, of which three plants were homozygous *alal* while the other four were heterozygous *Alal* (Figure 2, Table 2). Two of the *alal* homozygous self-fertile F_2_ plants have the A-type cytoplasm, and the third has the T-type cytoplasm. These results are in line with the hypothesis that the homozygous *alal* genotype results in male sterility in the P cytoplasm type but not in T and A cytoplasm.

To provide further evidence for the male fertility of *alal* plants with A and T cytoplasm, we planted five tubers each from four *alal* F_2_ plants with A cytoplasm and two *alal* F_2_ plants with the T cytoplasm. In addition, we sowed F3 seed from seven self-fertile F_2_ plants (two with A cytoplasm and fixed for the *al* allele and five with T cytoplasm of which four are fixed for the *al* allele, and two of which segregate for *Al* and *al* (Table 2)). We genotyped the tuber-grown BC_2_F_2_ plants, as well the TPS-grown F_3_ seedlings with the same KASP markers that we used in the TPS-raised BC_2_F_2_ populations. All tuber-grown F_2_ plants and F_3_ populations derived from *alal* F_2_ plants were confirmed to be *alal*, whereas the F_3_ populations derived from *Alal* heterozygous F_2_ plants segregated for the *Al* locus. Interestingly, the BC_2_F_2_ plants raised from tubers from genotypes BC2F2(A)-1-29, BC2F2(A)-2-44 and BC2F2(T)-3-16 showed improved self-fertility compared to the same genotypes raised from TPS, suggesting improved vigor of the tuber-raised plants compared to seedling-raised plants (Table 3). The F_3_ populations suffered from inbreeding depression showing overall poor fertility. Nevertheless, seven *alal* homozygous F3 plants with A cytoplasm and six *alal* homozygous F_3_ plants with T cytoplasm produced berries and seed upon self-pollination, ranging from 15 to 297 seeds per plant (Table 4).

Taken together, the apparent male fertility of the tuber-raised *alal* homozygous F_2_ plants and the seedling-raised *alal* homozygous F_3_ plants provide clear evidence that homozygosity for the *al* allele does not result in male sterility in T and A cytoplasm, providing convincing evidence that the antherless trait is a form of cytoplasmic male sterility.

### 3.4. Application and Deployment of the Antherless Gene

**Figure 3:**
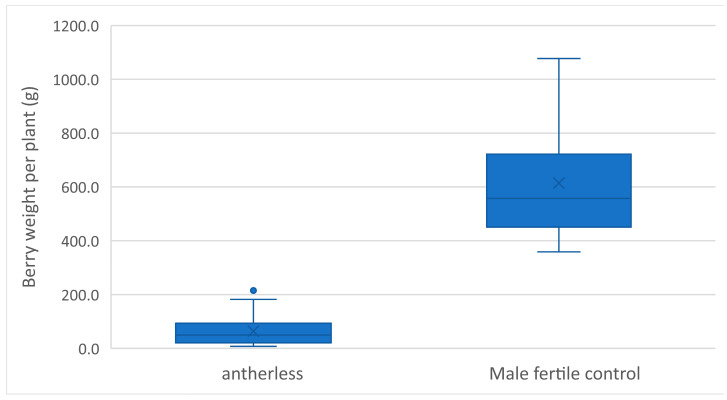
Berry yield of antherless POC hybrids and male fertile controls in a field trial. Antherless hybrids (n = 26) produce significantly fewer berries than male fertile controls (n = 8) (*p* < 0.001). The boxes represent the 1st and 3rd quartiles, the horizontal lines in the box represent the medians, and the × represent the means.

With the antherless CMS system, an improved inbred line-based hybrid breeding system becomes feasible, whereby male sterile inbred lines and hybrids are generated. After an initial cross between an elite inbred line with P cytoplasm and an *al* donor line, markers can be used to select heterozygous *Alal* progeny after the initial cross and after each round of self-fertilization or backcrossing. When a sufficient level of homozygosity has been achieved, a final round of self-fertilization can be performed and homozygous *alal* progeny can be selected using the same markers, resulting in a male sterile maternal inbred line. Similarly, after an initial cross between an elite line with T or A cytoplasm and an *al* donor, the markers can be used to select for heterozygosity during backcrossing or homozygosity during inbreeding, resulting in male fertile *alal* inbred lines. With this system, emasculation of the maternal lines is no longer necessary and hybridization will result in male sterile F_1_ hybrids. To determine the feasibility of this system, we fertilized five male sterile *alal* homozygous lines in P cytoplasm with pollen from four male fertile *alal* homozygous lines with A or T cytoplasm and obtained seeds from 13 crosses (Table S8). Next, we planted these 13 male sterile hybrids together with male fertile control hybrids in a field trial and harvested all berries at the end of the growing season. While the male sterile antherless hybrids did set some berries, the total be yield was significantly lower than those of male fertile control hybrids (Figure 3).

## 4. Discussion

Diploid hybrid potato breeding is based on the generation of homozygous inbred parent lines by continuous self-fertilization and hybrid cultivars by crossing these parent lines. For inbreeding, self-fertilized berry set is crucial, while this is an undesired character for the cultivation of potato in the field. In this study, we investigated the antherless CMS system which can be used to generate potato hybrids that set few berries in the field. We identified a recessive allele (*al*) on the short arm of Chromosome 6 of *S. microdontum* subsp. *gigantophyllum* which results in complete male sterility due to malformed or absent anthers in the presence of the P cytoplasm (Figure 1a).

Previously, Endelman and Jansky identified the *Sa* locus, involved in male sterility, which was mapped to the short arm of Chromosome 6 in an F_2_ population from the cross between DM and M6. The recessive allele *sa* originates from the M6 parent, and the short anther phenotype is likely a result of an interaction between the *Sa* locus and the P cytoplasm from DM [43]. Given the similarity between the antherless and short anther traits of the *Al* and the *Sa* locus, it is possible that these loci are identical. However, as both loci originate from distant *Solanum* species, these loci might also be different. Therefore, here we use the designation “*Al* locus” to refer to the *antherless* locus from *S. microdontum* subsp. *gigantophyllum*

In this study, we have found that the antherless trait is expressed in P, but not in T and A cytoplasm, proving that antherless is a form of CMS and that it is the result of an interaction between the nuclear genome and the cytoplasm. It is likely that the dominant allele of the *Al* locus encodes a fertility restorer that is required for fertility in genotypes carrying the P cytoplasm. Unfortunately, our efforts to fine-map the *Al* locus were hindered by a lack of recombination in the region of the *Al* locus. Among 2011 BC_2_ individuals, we did not find any true recombinants in the 25.12 Mb interval on the short arm of Chromosome 6 (Table S3). The suppression of recombination in this interval on Chromosome 6 is observed in many potato genetic maps, suggesting that this region is pericentromeric and that fine-mapping approaches will not reduce the size of the interval [16,43,54,55,56,57]. Thus, identification of the causal gene requires an alternative strategy. One strategy would be to perform untargeted mutagenesis on seed from a population derived from a cross between an *alal* and an *AlAl* individual wherein the maternal parent has the P cytoplasm. All progeny from such a cross are heterozygous *Alal* and should have normal male fertile anthers. However, when the dominant *Al* allele of such a genotype is knocked out, it results in an *alal* genotype, which is antherless and can easily be identified among the fertile plants of this backcross population. The causal mutation can then be identified by comparing sequence data from the mutant with the parental genomes. Such a strategy has been successful in the cloning of several genes from genomic regions with suppressed recombination and could be used to identify the *al* allele [58,59,60]. Alternatively, a targeted mutagenesis approach could reveal the causal gene for the antherless phenotype. This approach would rely on knowledge about nuclear Restorer of Fertility (Rf) genes involved in CMS in other crops and targeted knock-out of candidate genes using CRISPR-Cas. Multiple *Rf* genes have been identified, and the majority of those encode pentatricopeptide repeat (PPR) proteins [61,62]. PPR encoding genes have been implicated in restoration of fertility in crop species such as pepper [63], rice [64], radish [65,66], sorghum [67], soybean [68], cotton [69,70], and others. These *Rf-PPR* exert their function in mitochondria, where they bind CMS inducing mitochondrial mRNAs, leading to reduced levels of sterilizing protein via mRNA destabilization, modification or translation inhibition [64,71,72,73]. In potato, Anisimova et al. identified 38 sequence fragments with homology to *petunia Rf-PPR592* and *Capsicum annuum CaPPR6*. These fragments map to five genomic loci of the potato reference genome DM [74]. Interestingly two of the identified *Rf-PPR* genes are located within the interval of the *Al* locus and are good candidates as causal genes underlying the antherless and short anther traits.

The *Al* locus is located on the short arm of Chromosome 6 in a pericentromeric region where other floral traits like bud abortion and corolla shape mapped to as well. It is unclear whether the same causal genes are involved in anther malformation, bud abortion and corolla shape. It is not possible to remove any linked deleterious alleles from the interval due to the lack of recombination. However, in this study we did not observe obvious deleterious linkage drag associated with the antherless locus, but it is possible that linkage drag may reveal itself in more advanced materials and more detailed agronomical studies. We observed variation in the severity of anther malformation among the *alal* homozygous plants from population BC_2_(P)-1, but the genetic background of this variation remains unknown. The severity of anther malformation is relatively stable within individual plants and their clonal offspring, but genetic analysis within the *alal* homozygous group did not reveal any significant QTL (Table S5). It is possible that the combined effect of multiple segregating small-effect loci are responsible for the observed variation in anther malformation, although epigenetic control of such loci cannot be excluded. Further studies with large populations which are fixed for the *al* allele could resolve this question.

The implementation of CMS in hybrid potato breeding will provide two major advantages: (1) seed production becomes more efficient by eliminating the need to emasculate maternal flowers and (2) ware crop production will benefit from significantly reduced berry set on the field. In contrast to crops like maize and rice, where the seed are the commercial end product, implementation of CMS in potato does not require fertility to be restored in the hybrid, and hence a *three-line system* is redundant. Here, we show that the antherless CMS system can be used to develop *alal* male sterile maternal lines with P cytoplasm and male fertile *alal* lines with T or A cytoplasm and that the hybrids derived from crosses between these parental lines hardly set any berries in the field. Male sterile maternal *alal* lines can be generated by introduction of the *al* allele in the breeding germplasm and subsequent marker-assisted selection for *Alal* heterozygosity upon inbreeding. When such an inbred line has met the criteria for becoming a female parent, breeders can easily select *alal* homozygotes based on the clear anther phenotype and proceed with test crosses or commercial seed production. We observed a reduction in fertility upon inbreeding in the BC_2_F_2_ and BC_2_F_3_ populations; it is not clear to what extent this is caused by the cytoplasm type. While it is possible that T and A cytoplasm affect fertility upon inbreeding, the BC_2_F_2_ and BC_2_F_3_ populations were derived from crosses with non-inbred cytoplasm donors which likely contained significant recessive genetic load. This genetic load may have resulted in inbreeding depression and the associated reduction in fertility, which are commonly observed during inbreeding of potato, especially in early inbred generations [75,76,77,78]. So, we successfully inbred *alal* lines with T and A cytoplasm up to the BC_2_F_4_ generation, used the *alal* BC_2_F_2_ as male parents in hybrid crosses with male sterile *alal* lines and showed that the resulting hybrids produce far fewer berries than male fertile controls. However, the antherless hybrids did still set some berries, likely caused by insect or wind-driven cross-pollination. Many male fertile diploid potato genotypes were present in and around our field experiment, providing ample fertile pollen which could have fertilized the antherless hybrids. In future commercial application of the antherless CMS system, the produced CMS hybrids will likely be grown in fields without male fertile diploid pollen donors where the CMS hybrids would likely set fewer or no berries at all. We consider this an effective proof of principle of the antherless CMS system. Further research is needed to determine how inbreeding depression in T and A cytoplasm affects fertility and plant vigor and whether linkage drag is an issue for the *Al* locus. Future research may also focus on identification of the causal gene and several good leads are available.

## 5. Conclusions

In conclusion, in this study we identified the recessive *al* locus on chr06 that causes male sterility in diploid potato with P cytoplasm but not in A and T cytoplasm. Efforts to more precisely localize the locus were not successful due to a lack of recombination in this region of chr06. We implemented the antherless CMS system in a hybrid breeding scheme and show that the resulting F_1_ hybrid set significantly fewer berries under field conditions. Further exploration of this trait could focus on the identification of the causal nuclear gene via a candidate gene approach and CRISPR-Cas-induced knock-out, as well as the identification of the corresponding mitochondrial or chloroplast gene.

## Figures and Tables

**Table 1:** Segregation distortion in F_2_ populations with T and A cytoplasm.

Population	*Antherless* Genotype			χ2	*p*-Value
	*AlAl*	*Alal*	*alal*		
BC2F2(A)-1	33	38	6	18.95	0.00008
BC2F2(A)-2	23	34	7	8.25	0.01616
BC2F2(T)-6	16	21	8	3.04	0.21823
BC2F2(T)-1	12	23	10	0.2	0.90484
BC2F2(T)-2	10	19	9	0.05	0.97404
BC2F2(T)-3	11	22	11	0	1
BC2F2(T)-4	8	31	20	5.03	0.08071
BC2F2(T)-5	1	45	39	34.27	0.00001

**Table 2:** Fertility characteristics of eight self-fertile F2 genotypes and their genotype at the *Al* locus.

Genotype	Cytoplasm Type	# Observed Flowers	Anther Phenotype (0–3)	Pollen Shed (0–3)	# Self-Berries	# Spontaneous Berries	# Seeds	*Antherless* Genotype	F3 Population
BC2F2(A)-1-29	A	20	3	3	5	2	23	*alal*	BC2F3(A)-1
BC2F2(A)-2-44	A	11	2	2	3	0	305	*alal*	BC2F3(A)-2
BC2F2(T)-2-07	T	23	3	1	2	1	106	*Alal*	BC2F3(T)-1
BC2F2(T)-2-20	T	25	3	3	5	2	220	*Alal*	BC2F3(T)-2
BC2F2(T)-3-06	T	6	3	1	3	0	97	*Alal*	BC2F3(T)-3
BC2F2(T)-4-16	T	30	3	3	10	16	140	*alal*	BC2F3(T)-4
BC2F2(T)-4-17	T	14	2	3	2	0	6	*Alal*	
BC2F2(T)-6-02	T	27	2	1	2	1	9	*Alal*	BC2F3(T)-5

**Table 3:** Flowering, berry and seed set of tuber-grown BC_2_F_2_ genotypes.

Genotype	Cytoplasm Type	Antherless Genotype	# Flowers	Anther Phenotype	Pollen Shed (0–3)	# Selfings	# Berries	# Seeds	Pollen Viability
BC2F2(A)-1-29-C1	A	*alal*	11	3	1-2	6	4	1296	0.9
BC2F2(A)-1-29-C2	A	*alal*	7	3	1	4	2	135	0.9
BC2F2(A)-1-29-C3	A	*alal*	17	3	1	4	1	22	0.9
BC2F2(A)-1-29-C4	A	*alal*	9	3	1-2	3	3	4	0.9
BC2F2(A)-1-29-C5	A	*alal*	11	3	1-2	1	1	49	0.9
BC2F2(A)-2-09-C1	A	*alal*	17	2-3	1	6	26	12	0.8
BC2F2(A)-2-09-C2	A	*alal*	0	N.D	N.D	0	N.D	N.D	N.D
BC2F2(A)-2-09-C3	A	*alal*	3	3	1	1	13	1	0.8
BC2F2(A)-2-09-C4	A	*alal*	7	2-3	1-2	2	25	0	0.8
BC2F2(A)-2-09-C5	A	*alal*	11	2-3	1	1	12	3	0.8
BC2F2(A)-2-39-C1	A	*alal*	4	2	0	0	N.D	N.D	N.D.
BC2F2(A)-2-39-C2	A	*alal*	3	2	0	0	N.D	N.D	N.D.
BC2F2(A)-2-39-C3	A	*alal*	0	N.D	N.D	0	N.D	N.D	N.D.
BC2F2(A)-2-39-C4	A	*alal*	1	2	0	0	N.D	N.D	N.D.
BC2F2(A)-2-39-C5	A	*alal*	0	N.D	N.D	0	N.D	N.D	N.D.
BC2F2(A)-2-44-C1	A	*alal*	32	2-3	3	17	19	740	0.9
BC2F2(A)-2-44-C2	A	*alal*	32	2-3	3	24	28	2457	0.9
BC2F2(A)-2-44-C3	A	*alal*	11	2-3	3	10	23	2320	0.9
BC2F2(A)-2-44-C4	A	*alal*	15	2-3	3	12	19	719	0.9
BC2F2(A)-2-44-C5	A	*alal*	24	2-3	3	13	34	1246	0.9
BC2F2(T)-3-07-C1	T	*alal*	17	3	1	4	2	18	N.D.
BC2F2(T)-3-07-C2	T	*alal*	20	3	1	6	3	40	N.D.
BC2F2(T)-3-07-C3	T	*alal*	24	3	0-1	3	2	3	N.D.
BC2F2(T)-3-07-C4	T	*alal*	33	3	0-1	8	4	82	N.D.
BC2F2(T)-3-07-C5	T	*alal*	38	3	0-1	8	7	75	N.D.
BC2F2(T)-4-16-C1	T	*alal*	52	3	3	16	21	550	0.9
BC2F2(T)-4-16-C2	T	*alal*	47	3	3	17	21	906	0.9
BC2F2(T)-4-16-C3	T	*alal*	40	3	3	10	7	177	0.9
BC2F2(T)-4-16-C4	T	*alal*	44	3	3	14	10	199	0.9
BC2F2(T)-4-16-C5	T	*alal*	43	3	3	20	37	843	0.9

**Table 4:** Flowering, berry and seed set of TPS-grown BC_2_F_3_ genotypes.

Genotype	Parent	Cytoplasm Type	Antherless Genotype	# Flowers	Anther Phenotype (0–3)	Pollen Shed (0–3)	# Selfings	# Berries	# Seeds	Pollen Viability
BC2F3(A)-1-11	BC2F2(A)-1-29	A	*alal*	21	3	1	5	5	15	N.D.
BC2F3(A)-1-12	BC2F2(A)-1-29	A	*alal*	12	3	3	9	3	48	N.D.
BC2F3(A)-2-04	BC2F2(A)-2-44	A	*alal*	9	2	2	7	14	98	N.D.
BC2F3(A)-2-05	BC2F2(A)-2-44	A	*alal*	2	2	3	2	1	125	N.D.
BC2F3(A)-2-14	BC2F2(A)-2-44	A	*alal*	5	2	3	5	3	242	85%
BC2F3(A)-2-17	BC2F2(A)-2-44	A	*alal*	7	2	3	7	10	278	90%
BC2F3(T)-2-06	BC2F2(T)-2-20	T	*AlAl*	5	3	3	5	4	83	60%
BC2F3(T)-2-09	BC2F2(T)-2-20	T	*Alal*	9	3	2-3	8	5	30	70%
BC2F3(T)-2-24	BC2F2(T)-2-20	T	*Alal*	13	3	2-3	18	11	336	70%
BC2F3(T)-3-27	BC2F2(T)-3-06	T	*Alal*	22	2	3	18	12	1514	90%
BC2F3(T)-3-29	BC2F2(T)-3-06	T	*Alal*	45	3	1-2	22	21	707	90%
BC2F3(T)-4-01	BC2F2(T)-4-16	T	*alal*	35	2-3	2-3	24	10	55	85%
BC2F3(T)-4-07	BC2F2(T)-4-16	T	*alal*	32	3	2-3	19	1	10	60%
BC2F3(T)-4-13	BC2F2(T)-4-16	T	*alal*	15	3	3	24	21	122	70%
BC2F3(T)-4-25	BC2F2(T)-4-16	T	*alal*	17	3	1-2	7	10	59	60%
BC2F3(T)-4-31	BC2F2(T)-4-16	T	*alal*	49	3	2	23	11	69	N.D.
BC2F3(T)-5-02	BC2F2(T)-4-16	T	*alal*	22	3	3	19	13	297	90%

## Data Availability

All data are contained within the article or Supplementary Materials.

## Supplementary Materials

The following supporting information can be downloaded at: https://www.mdpi.com/article/10.3390/biology13060447/s1, Figure S1: Cytoplasm type determination using multiplex PCR markers as described by Hosaka and Sanetomo; Table S1: Plant materials used in this study; Table S2: Genotypic data of population BC2(P)-1; Table S3: Genetic map of population BC2(P)-1; Table S4: Phenotypic data from population BC2(P)-1; Table S5: QTL identified in population BC2(P)-1; Table S6: Genotypic data from putative recombinants from population BC2(P)-2; Table S7: Phenotypic data from BC2F2 populations with A and T cytoplasm; Table S8: Berry weights per plant of antherless and male fertile control plants.
